# Anomaly-resilient geofencing and predictive navigation in IoT environments using machine learning and federated learning for metaverse workplaces and smart shopping malls

**DOI:** 10.1038/s41598-025-33856-0

**Published:** 2026-01-19

**Authors:** Noor El-Deen M. Mohamed, Mahmoud A. Shafea, Mostafa M. Abdelhakam

**Affiliations:** 1 Computer and Systems Engineering Department, Faculty of Engineering, Helwan University, Cairo, Egypt; 2 Electronics and Communications Engineering Department, Faculty of Engineering, Helwan University, Cairo, Egypt

**Keywords:** Engineering, Mathematics and computing

## Abstract

The rapid convergence of physical and digital environments is redefining user interactions in both professional and retail sectors. While the concept of the Metaverse offers new avenues for immersive remote collaboration, complex physical venues such as shopping malls require intelligent optimization to mitigate navigational inefficiencies and enhance user satisfaction. This research integrates augmented reality (AR), virtual reality (VR), and the Metaverse alongside machine learning (ML) and Federated Learning (FL) to create virtual spaces for workplace meetings in the Meta Workplaces Monitoring System (MetaWMS) and an active navigation application for shopping malls, the Meta Shopping Navigation System (MetaSNS). To ensure data integrity within these IoT environments, anomaly detection is applied prior to geofencing to filter out spurious Wi-Fi network signatures, such as mobile hotspots. Validated against the Aegean Wi-Fi Intrusion Dataset 3 (AWID3), the proposed One-Class SVM gatekeeper achieves a detection accuracy of 93.5%, significantly outperforming KNN (86.6%) and Isolation Forest (67.4%). Geofencing is then used to define virtual perimeters, enabling location-specific AR experiences. Building on previous work in indoor geofence detection, this paper extends the framework to support intelligent navigation using the large-scale Microsoft Research Indoor Location dataset. Sequence Prediction is performed using a Long Short-Term Memory (LSTM) architecture to forecast users’ next likely destinations, achieving prediction accuracies of 59%, 77%, and 83% for top-1, top-3, and top-5 recommendations, respectively. To preserve privacy, Federated Learning (FL) is employed so that only model weights, rather than raw data, are shared with the server, introducing a marginal accuracy loss of 2–5% while ensuring privacy-preserving personalization.

## Introduction

The integration of the Metaverse and Internet of Things (IoT) into physical infrastructure represents a paradigm shift in how individuals interact with professional and retail environments. In the corporate sector, organizational structures have been redefined by the evolution of hybrid work models accelerated significantly by the global pandemic often creating a disconnect that hinders spontaneous collaboration^1,2^. Simultaneously, physical retail venues like shopping malls face increasing pressure to provide seamless, personalized user experiences comparable to digital platforms^3^. Addressing these evolving demands requires intelligent systems capable of bridging the physical and virtual worlds leveraging Augmented Reality (AR) to enhance connectivity in workplaces and navigational efficiency in shopping malls.

However, realizing this vision of intelligent, location-aware environments is hindered by two fundamental challenges. First, indoor positioning using ubiquitous Wi-Fi infrastructure is plagued by signal instability and environmental noise. In dynamic public spaces, rogue signals from transient sources such as personal mobile hotspots can corrupt location fingerprints, leading to significant positioning errors^4,5^. Second, transforming navigation from a reactive service to a proactive one requires predicting user intent. Yet, analyzing sensitive movement trajectories to forecast future destinations raises critical privacy concerns, discouraging user adoption in surveillance-wary ecosystems^6^.

To overcome these barriers, this research hypothesizes that a robust, privacy-preserving navigational framework can be established by filtering environmental noise before classification and maintaining sensitive data on-edge. A unified ecosystem is proposed that integrates anomaly detection, deep learning-based sequence prediction, and Federated Learning (FL). Unlike traditional approaches that rely on centralized data aggregation, this framework ensures that raw user trajectories never leave the device.

The main objectives and novel contributions of this work are established through four key developments. First, a pre-processing gatekeeper module utilizing a One-Class Support Vector Machine (SVM) is introduced to ensure data integrity. This component effectively filters spurious Wi-Fi signatures before they reach the classifier, achieving an anomaly detection accuracy of 93.5% and an Area Under the Curve (AUC) of 0.945 on real-world datasets. Second, a Long Short-Term Memory (LSTM) network is implemented to proactively forecast a user’s next likely destination based on their immediate trajectory. This approach achieves a Top-5 prediction accuracy of 80.59% in complex retail environments, significantly enhancing the user experience compared to reactive positioning alone. Third, data privacy is addressed through a Federated Learning implementation using the FedAvg algorithm. By exchanging only model weight updates rather than raw location history, the system preserves user privacy with a marginal accuracy trade-off of only 2–5% compared to centralized training. Finally, a holistic system architecture is presented that seamlessly couples these machine learning backend components with frontend AR/VR interfaces, validated through functional prototypes for both remote workplace monitoring and mall navigation.

## Related work

This section reviews recent advancements in the three core domains underpinning the MetaSNS framework: anomaly detection in wireless signals, sequence prediction for indoor mobility, and privacy-preserving federated learning. A comparative summary of key literature is provided in Table 1.

### Wi-Fi anomaly detection in IoT

The reliability of indoor positioning systems (IPS) is fundamentally constrained by the volatility of Received Signal Strength Indicator (RSSI) values. Recent studies have focused on machine learning approaches to filter environmental noise and ”rogue” signal sources. Alsaadi et al. (2025) conducted a comparative analysis of unsupervised algorithms for IoT security, demonstrating that One-Class Support Vector Machines (OC-SVM) achieve superior precision and recall compared to Isolation Forest (IF) when detecting point anomalies in high-dimensional sensor data^7^. Similarly, Dai (2025) evaluated density-based methods against boundary-based methods, concluding that while Isolation Forest is computationally efficient, SVM-based approaches offer greater robustness in defining clear decision boundaries for normal traffic patterns^8^. These findings support the hypothesis that boundary-based classifiers are better suited for filtering transient Wi-Fi hotspots than tree-based isolation methods, which may struggle with the high variance of legitimate RSSI fluctuations^5^.

### Sequence prediction for indoor mobility

Predicting user trajectories in GPS-denied environments has evolved from probabilistic Markov models to deep learning architectures. While Transformer-based models have gained prominence for their ability to capture long-range dependencies, recent work by Smith and Johnson (2025) notes that Transformer encoders can be computationally prohibitive for real-time edge deployment on mobile devices^9^. In contrast, Recurrent Neural Networks (RNNs), specifically Long Short-Term Memory (LSTM) networks, remain the standard for resource-constrained sequence modeling. Zhang and Wang (2025) demonstrated that LSTM architectures effectively mitigate signal instability in visible light positioning, achieving robust next-step prediction with significantly lower latency than attention-based alternatives^10^. Furthermore, Song (2023) highlighted that lightweight LSTMs with optimized window sizes can achieve high predictive accuracy on edge devices, balancing the trade-off between model complexity and energy consumption^11^.

### Federated learning in indoor navigation

The shift towards privacy-preserving computing has driven the adoption of Federated Learning (FL) in location-based services. Jan et al. (2024) proposed a hierarchical FL system for indoor localization, demonstrating that decentralized model training can achieve accuracy comparable to centralized approaches while significantly reducing bandwidth usage^12^. More recently, Gufran and Pasricha (2023) introduced **FedHIL**, a framework designed to handle device heterogeneity in indoor localization^13^. FedHIL utilizes a selective weight adjustment mechanism to mitigate the impact of noisy client data, achieving state-of-the-art results in coordinate regression (minimizing positioning error). However, challenges remain regarding the computational overhead of such regression models on edge devices. Liu and Chen (2025) addressed this by integrating adaptive transfer learning^14^, though standard implementations like Federated Averaging (FedAvg)^15^ continue to offer the most reliable balance of communication efficiency for real-time mobile ecosystems.

### Research gap and motivation

Despite these advancements, significant gaps remain in the integration of these technologies. As summarized in Table 1, most existing works address these challenges in isolation. Anomaly detection studies^7,8^ primarily focus on network security (Intrusion Detection Systems) rather than signal integrity for localization. Similarly, while deep learning models for trajectory prediction exist^9,10^, they rarely account for the privacy implications of centralizing user movement data. Conversely, privacy-focused studies^12^ often rely on simulated datasets that fail to capture the noisy reality of public retail environments.

This research addresses these gaps by proposing a unified, edge-deployable framework that: (1) adapts OC-SVM specifically for Wi-Fi signal cleaning, (2) utilizes a lightweight LSTM for proactive guidance, and (3) integrates these within a Federated Learning architecture to ensure privacy-preserving personalization in real-world scenarios.

**Table 1:** Comparative analysis of related literature (2023-−2025).

**Approach/Framework**	**Year**	**Primary focus**	**Methodology**	**Limitations addressed by this work**
FedHIL^13^	2023	Coordinate Localization	Selective Weight Adjustment	Focuses on regression (RMSE), higher compute cost.
Lightweight LSTM^11^	2023	Sequence Prediction	Hyperparameter Optimization	Lacks privacy preservation (Centralized).
Hierarchical FL^12^	2024	Privacy	Hierarchical Architecture	Focuses on localization accuracy, not predictive guidance.
IoT Anomaly IDS^7^	2025	Anomaly Detection	Comparison of IF vs. OC-SVM	Domain is IoT Security (IDS), not Indoor Positioning.
Transformer Encoder^9^	2025	Trajectory Recovery	Attention Mechanisms	High computational cost for mobile edge deployment.
**MetaSNS (Proposed)**	**2025**	**Holistic Navigation**	**SVM + LSTM + FedAvg**	**Integrates anomaly filtering, prediction, and privacy.**

## System design and architecture

This research proposes a unified ecosystem comprising two complementary frameworks: MetaWMS for remote workplace monitoring and MetaSNS for predictive shopping navigation. Both frameworks share a common architectural backbone consisting of a central web platform, mobile sensing clients, and immersive AR/VR interfaces.

### Evolution from conference version

This work represents a substantial extension of the preliminary MetaWMS framework presented in^16^. While the conference version focused exclusively on reactive monitoring within structured workplace environments using a basic Artificial Neural Network (ANN), this study introduces the MetaSNS framework to address the dynamic and noisy nature of public spaces, specifically shopping malls.

The system architecture has been fundamentally redesigned to transition from a static location detection service to a proactive, privacy-preserving navigational ecosystem. Key advancements include the integration of an anomaly detection ”gatekeeper” to filter spurious signal noise, the implementation of a Long Short-Term Memory (LSTM) network for next-step trajectory prediction, and the adoption of a Federated Learning (FL) architecture to decentralize model training. Furthermore, the experimental scope has been expanded from a single proprietary campus dataset to include three diverse public datasets (AWID3, StudentLife, and Microsoft Research Indoor Location), ensuring robust validation against real-world variability. Table 2 provides a detailed comparison between the initial conference implementation and the current journal version.

**Table 2:** Comparison of contributions: conference version vs. journal version.

**Feature/Aspect**	**Conference version (MetaWMS) **^16^	**Current journal version (MetaSNS)**
**Domain Scope**	Remote Workplaces (Static)	Workplaces + Shopping Malls (Dynamic)
**Data Integrity**	None (Raw Wi-Fi input)	**Anomaly Detection Module** (OC-SVM) to filter hotspots
**Navigation Logic**	Reactive (Detects current zone only)	**Proactive** (Predicts next likely zone using LSTM)
**Privacy Architecture**	Centralized (Server-side processing)	**Federated Learning** (On-device training, FedAvg)
**Experimental Data**	Proprietary Campus Dataset (Helwan Univ.)	Campus + **AWID3** + **Indoor Location & Navigation**
**Localization Output**	Binary Presence (Inside/Outside)	Semantic Trajectory Prediction (Top-*k* Ranking)

### Core ecosystem and web platform

**Fig. 1:**
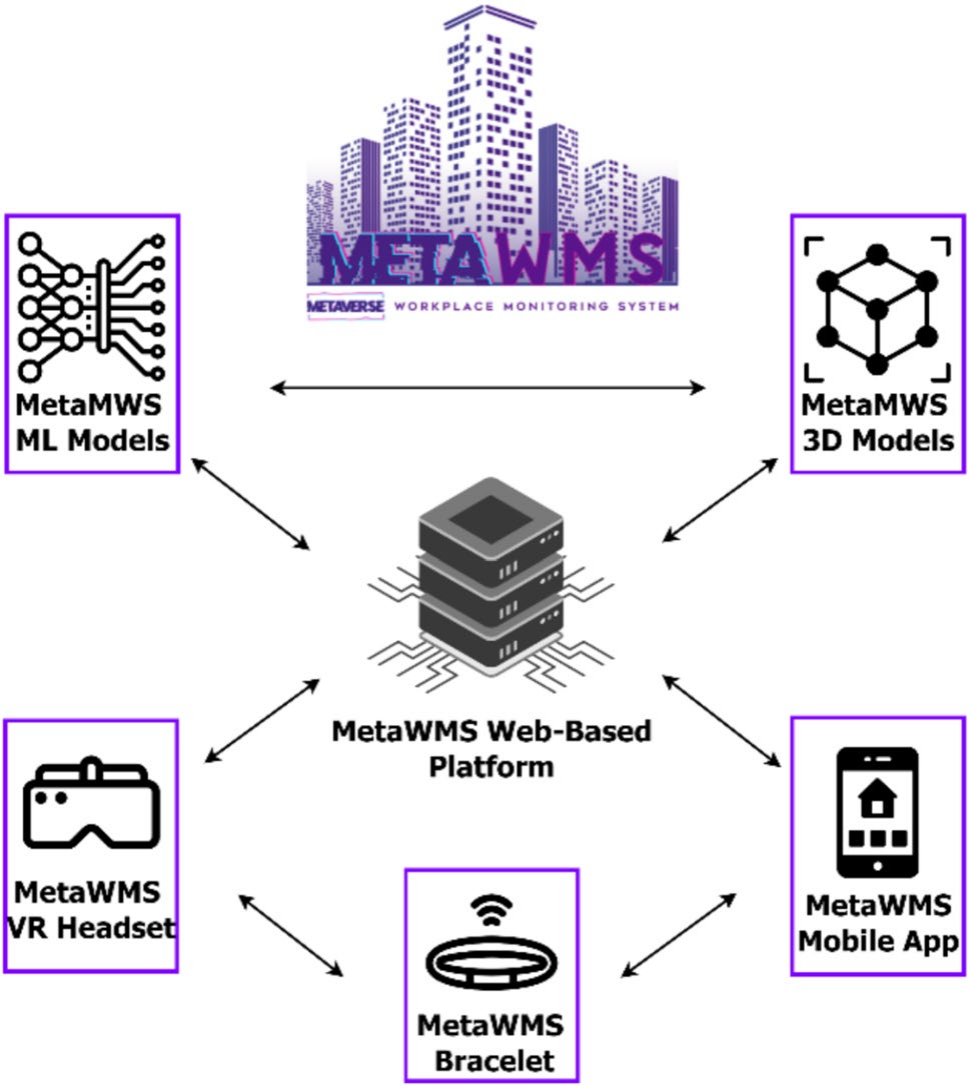
MetaWMS system architecture.

The original MetaWMS (Metaverse Workplace Monitoring System) architecture, as presented in^16^, addresses the critical need for seamless connectivity in remote work environments. It integrates geofencing and immersive technologies to enable employees to interact as if physically co-located, a concept aligned with previous studies^17^. At the core of this architecture is a web-based platform developed using React.JS, PHP, and Laravel, acting as a central hub as depicted in Fig. 1.

This platform orchestrates real-time monitoring and AR environment loading by integrating machine learning (ML) models that process Wi-Fi BSSID and RSSI values. Based on user-specific indoor geofence detection, the system dynamically loads 3D models representing workplace environments (e.g., meeting rooms). A primary function of this web system is the storage and delivery of these 3D assets with the assistance of Zappar services^18^, enabling on-demand AR rendering only when a user is confirmed within a valid geofence. The platform also manages the categorization of geofences into ”active” (GPS-based for outdoors) and ”passive” (Wi-Fi ML-based for indoors), providing a dashboard for administrators to monitor attendance and activity logs.

### Mobile applications and user interface

The system relies on specialized mobile applications to bridge the physical and virtual worlds. These applications function as sensor hubs for data collection and rendering engines for immersive content.

#### Workplace interface (MetaWMS)

**Fig. 2:**
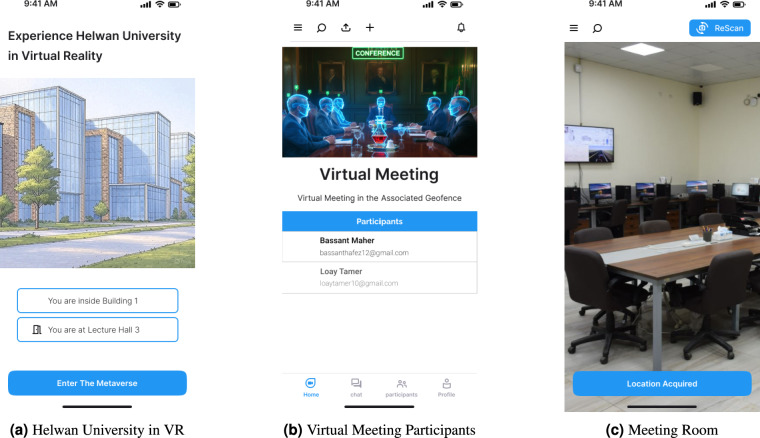
Overview of the workplaces application components.

A primary application of the framework is the augmentation of physical workspaces with a digital metaverse layer. To avoid the computational burden of storing extensive 3D models locally, MetaWMS employs an optimized, on-demand, server-side rendering architecture. The user interaction flow begins when the mobile application identifies the user’s presence within a registered location (e.g., a home office). The interface then presents the option to “Enter The Metaverse,” as illustrated in Fig. 2a. Upon initiation, the mobile device authenticates with the server and streams back the corresponding virtual environment.

To further enhance the sense of immersion, the system integrates with virtual reality headsets. By leveraging a 110-degree field of view, the headset provides a 360-degree virtual reality experience that closely simulates physical presence within the digital workspace. This capability enables organizations to provide virtual workspaces that can be accessed from any geographic location, bridging the gap between disparate physical environments and a unified virtual office.

#### Shopping navigation interface (MetaSNS)

The shopping mall navigation application functions as a sophisticated sensor hub, tasked with achieving real-time, sub-meter localization accuracy in GPS-denied indoor environments^19^. The mobile client employs sensor fusion techniques utilizing the magnetometer, Wi-Fi transceivers, barometer, and gyroscope/accelerometer for Pedestrian Dead Reckoning (PDR).

**Fig. 3:**
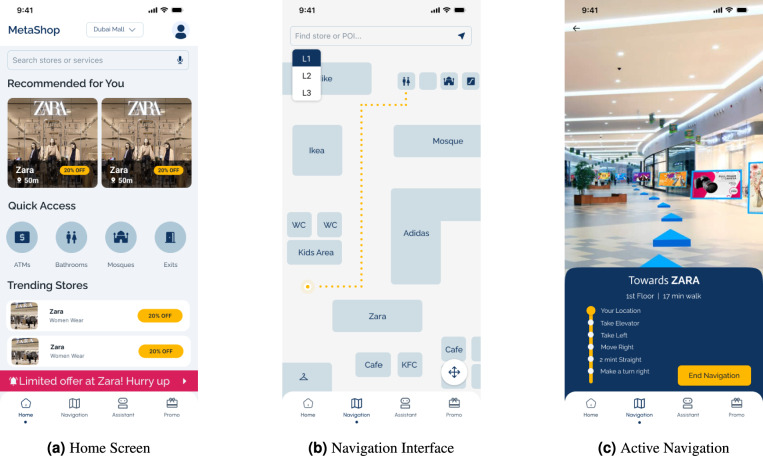
Overview of the shopping application components.

Crucially, the mobile application manages the triggering of location-based AR offers. Geofences around stores are synchronized from the backend; when the client detects a geofence breach, it triggers the retrieval of associated 3D AR assets, such as interactive discount overlays. This mechanism facilitates personalized and high-impact retail promotions, leveraging the immersive nature of AR to boost shopper engagement^20^. Figure 3a, b, and c illustrate the user journey from the home dashboard to active AR navigation.

### Enhanced machine learning pipeline

While the core architecture handles connectivity and rendering, the intelligence of the system relies on a multi-stage machine learning pipeline designed to address signal noise and data privacy. This pipeline introduces two critical advancements: a data integrity stage and a proactive sequence prediction module.

#### Data integrity and anomaly detection

**Fig. 4:**
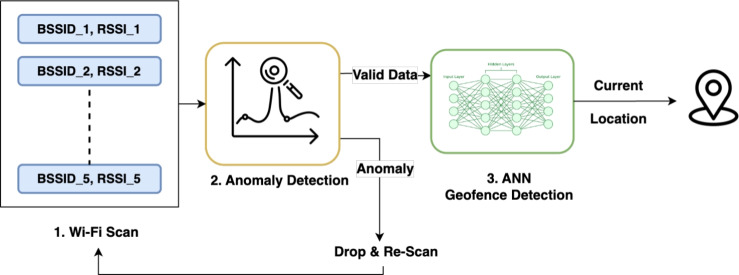
Anomaly detection before getting location.

The first stage functions as a gatekeeper to the core positioning system. The use of machine learning for outlier detection is a well-established approach for ensuring data integrity in various sensor-based systems, including safety-critical applications like pipeline inspection^21^. As illustrated in Fig. 4, raw Wi-Fi scans are immediately passed to an anomaly detection model which evaluates the scan’s fingerprint against a learned profile of normal signal patterns. If the reading is flagged as anomalous (e.g., a mobile hotspot), it is discarded. This iterative filtering process ensures that the subsequent geofence classifier is shielded from spurious data, significantly increasing reliability.

#### Proactive prediction and privacy

**Fig. 5:**
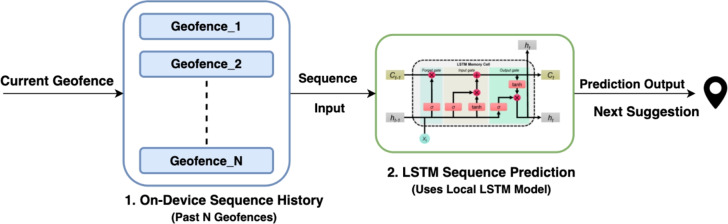
Sequence prediction.

**Fig. 6:**
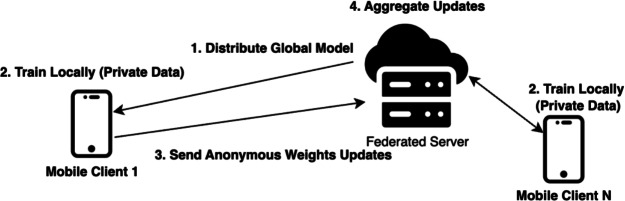
Federated learning process.

Following location determination, the system transforms from a reactive service into a proactive guide. As depicted in Fig. 5, a sliding window of visited locations is processed by a Long Short-Term Memory (LSTM) network to forecast the user’s next destination. To address privacy concerns commonly found in mobile sensing^6^, this model is trained using a Federated Learning (FL) framework (Fig. 6). Raw sequence data remains on the user’s device; only anonymized model weight updates are transmitted to the server for aggregation via the Federated Averaging (FedAvg) algorithm.

Algorithm 1 details this integrated client-side workflow, combining data acquisition, anomaly filtering, localization, and privacy-preserving updates.

**Algorithm 1:**
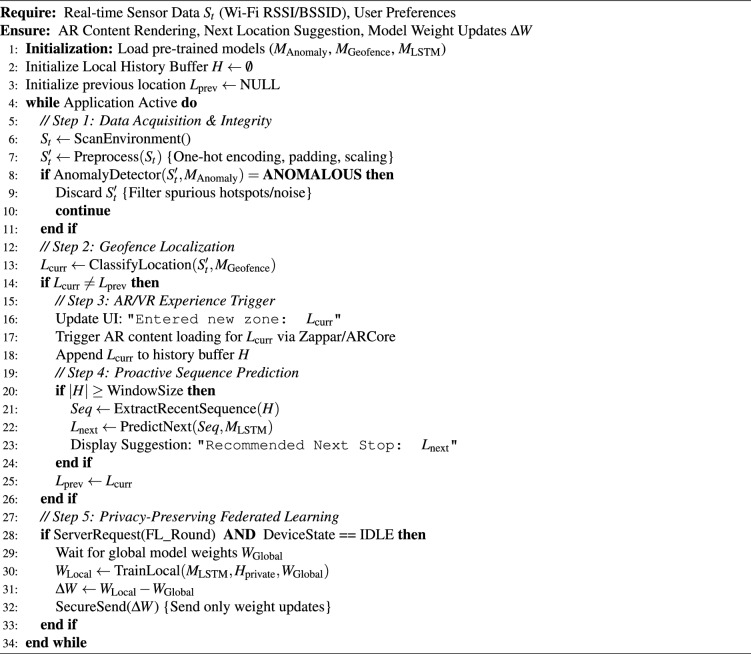
MetaSNS integrated client-side workflow

## Methodology and implementation details

This section details the specific datasets, preprocessing pipelines, and model hyperparameters utilized to implement the architecture described in Sect. "System Design and Architecture".

### Anomaly detection and geofence classification

#### Dataset and preprocessing

The implementation of this stage utilizes two primary data sources. The ”normal” dataset, containing legitimate Wi-Fi scans from fixed access points, is derived from the previous work in^22^, which fused Wi-Fi and magnetic field metrics for workplace monitoring. To evaluate the effectiveness of the filtering stage, this was supplemented by a synthetically generated ”anomalous” dataset, consisting of 100 rows designed to simulate the presence of personal mobile hotspots, characterized by unfamiliar BSSIDs and unusually strong RSSI values. A rigorous, multi-step preprocessing pipeline was implemented to prepare the raw sensor data for the machine learning models. First, to handle the categorical nature of BSSID values, which are incompatible with distance-based algorithms, The system employed (One-Hot Encoding). A vocabulary of all unique BSSIDs present in the ”normal” training dataset was created, and each BSSID was transformed into a sparse binary vector. Second, to ensure a fixed-size input for all models, each scan was padded to a uniform length corresponding to the top five BSSIDs and their RSSI levels. For scans detecting fewer than five access points, the missing BSSID vectors were represented by all zeros, and the corresponding RSSI values were padded with (−100 dBm). Finally, all numerical features the concatenated one-hot encoded vectors and the RSSI levels were standardized using the (StandardScaler) from the (scikit-learn) library^23^, which was fit exclusively on the ”normal” training data to prevent data leakage.

To address the limitations of synthetic anomalies and validate the system against realistic threats, the experimental framework was extended using the Aegean Wi-Fi Intrusion Dataset 3 (AWID3)^24^. This dataset represents a state-of-the-art benchmark for Wi-Fi security, collected in a physically isolated, real-world enterprise environment. It focuses on the IEEE 802.11ac and 802.11w standards, capturing over 25 million packets across 13 different attack classes using a diverse array of hardware targets (e.g., varying signal strengths and distances). For this study, the focus was placed on the ’Evil Twin’ and ’Rogue Access Point (AP)’ attack scenarios, as these closely mimic the transient mobile hotspot anomalies encountered in shopping malls. The raw packet data was transformed into a format compatible with the proposed system through a specialized preprocessing pipeline. Raw frames were grouped into 1-second time bins to simulate a device performing a standard Wi-Fi scan. Within each bin, the top 5 distinct Access Points were extracted based on signal strength. A scan was labeled as anomalous (−1) if it contained any packets marked as Evil Twin or Rogue AP; otherwise, it was labeled as normal (1). A critical enhancement in this stage was the feature scaling strategy. BSSIDs were first converted to numerical integers using a label encoder fitted only on normal traffic. Crucially, to prevent the large integer values of BSSIDs from dominating the distance metrics, a Min-Max Scaler was applied to both the encoded BSSIDs and the signal levels, normalizing all features to the range [0, 1]. The data was split such that the training set consisted exclusively of normal traffic (80% of normal samples), while the test set combined the remaining normal traffic with all anomalous samples to realistically evaluate detection capabilities.

#### Model implementation

To comprehensively evaluate the data filtering stage, three distinct anomaly detection algorithms were implemented using the ‘scikit-learn‘ library, each representing a different approach to outlier identification. Several approaches were investigated: a density-based approach with ”K-Nearest Neighbors (KNN)”^25^, a boundary-based approach with ”One-Class Support Vector Machine (SVM)”^26^, and an ensemble-based approach with ”Isolation Forest”^27^. Each model was trained solely on the preprocessed ”normal” dataset to learn the characteristics of legitimate Wi-Fi signals. The specific hyperparameters for each of the three anomaly detection models were chosen based on common practices in the literature to establish a robust baseline for comparison. For the K-Nearest Neighbors model, the number of neighbors (n_neighbors) was set to 5, defining the size of the local neighborhood used for density estimation. For the One-Class SVM, a Radial Basis Function (rbf) kernel was used to handle non-linear decision boundaries, and the (nu) parameter was set to 0.1 which acts as an upper bound on the fraction of training errors and serves as an estimate of the outlier proportion. For the Isolation Forest model, the number of estimators (n_estimators) was set to 100 to build a robust ensemble, and the contamination parameter was set to 0.1 to indicate the expected proportion of outliers, which assists the algorithm in setting its internal decision threshold. These parameters are summarized in Table 3.

**Table 3:** Hyperparameters for anomaly detection models.

**Model**	**Parameter**	**Value**
K-Nearest Neighbors	Number of neighbors	5
	Metric	Minkowski
One-Class SVM	Kernel	RBF
	Gamma	Auto
	Nu	0.1
Isolation Forest	Number of estimators	100
	Contamination	0.1

Following the anomaly detection filter, the geofence classification is performed by the same feedforward ”Artificial Neural Network (ANN)” architecture developed in the foundational work^16^. This lightweight network is designed for efficient on-device inference and consists of an input layer that accepts the preprocessed feature vector, two hidden layers with 10 and 8 neurons respectively using the ReLU activation function, and a final output layer with a single neuron and a sigmoid activation function. This output neuron produces a probability score indicating whether the user is inside (1) or outside (0) the target geofence. A key distinction in this enhanced pipeline is that the ANN only receives Wi-Fi network signatures that have been validated as ”normal” by the preceding anomaly detection module, thereby ensuring it operates on a cleaned and more reliable data stream. The trained model is subsequently converted for on-edge deployment on the mobile application using the TensorFlow Lite library^28^.

### Sequence prediction for proactive navigation

#### Dataset and preprocessing

The training and evaluation of the sequence prediction model were conducted using the StudentLife dataset, a large, longitudinal, and anonymized dataset collected from 48 undergraduate and graduate students over a 10-week academic term^29^. While the full dataset is rich with multimodal sensor data, including activity, sleep, and communication patterns, the implementation exclusively utilized the (wifi_location) data from 49 user files. This specific subset, initially comprising 1,893,838 rows, contains time-stamped geofence labels that form the basis for user movement trajectories. A comprehensive preprocessing pipeline was designed to transform this raw data into a structured format suitable for training a sequential model. The process began by loading the raw CSV files and structuring the data into a single DataFrame containing columns for timestamp, location, and a unique user ID. Following this, a series of cleaning and simplification steps were applied. Location strings were standardized (e.g., in[baker-berry] was simplified to baker-berry), and to focus on meaningful transitions between locations, consecutive duplicate entries for each user were removed. Furthermore, to reduce noise and the complexity of the prediction task, infrequent locations defined as those visited fewer than 10 times across the entire dataset were filtered out. This cleaning process was highly effective, reducing the approximately 9,000 initial unique location strings to a manageable vocabulary of 90, and condensing the dataset to 115,650 relevant location transitions.

To prepare the data for the LSTM, the cleaned location data for each user was segmented into distinct sessions. A session was defined as a continuous period of activity, with a new session beginning after a timeout period of two hours of inactivity. This step was crucial for ensuring that the model learned intra-session movement patterns and resulted in the identification of 5,642 valid sessions. The unique location labels were then tokenized by mapping each string to a unique integer ID, creating a final vocabulary of 91 classes (90 locations plus one padding token). From each session, input-output pairs were generated, where an input sequence of visited locations was used to predict the next location in the path. All input sequences were padded to a fixed length of 20 to create uniform tensors for model training. Finally, the generated sequences were split into training (86,826 sequences), validation (12,853 sequences), and test (10,110 sequences) sets.

To address the domain limitations of campus-based data and evaluate the system in realistic retail environments, this study further incorporates the Microsoft Research Indoor Location dataset^30^. Unlike the previous dataset, this source provides a large-scale, industry-standard benchmark for indoor positioning. The dataset encompasses 212 diverse buildings across three metropolitan cities, containing over 30,000 indoor walking traces and 100,000 Wi-Fi and Bluetooth iBeacon signatures. It provides rich sensing information, including aligned timestamps for accelerometer, gyroscope, and magnetometer readings, alongside human-labeled ground truth for varying floor plans. The preprocessing pipeline was adapted to handle this continuous spatial data by focusing on the top 10 sites with the highest volume of path files.

**Fig. 7:**
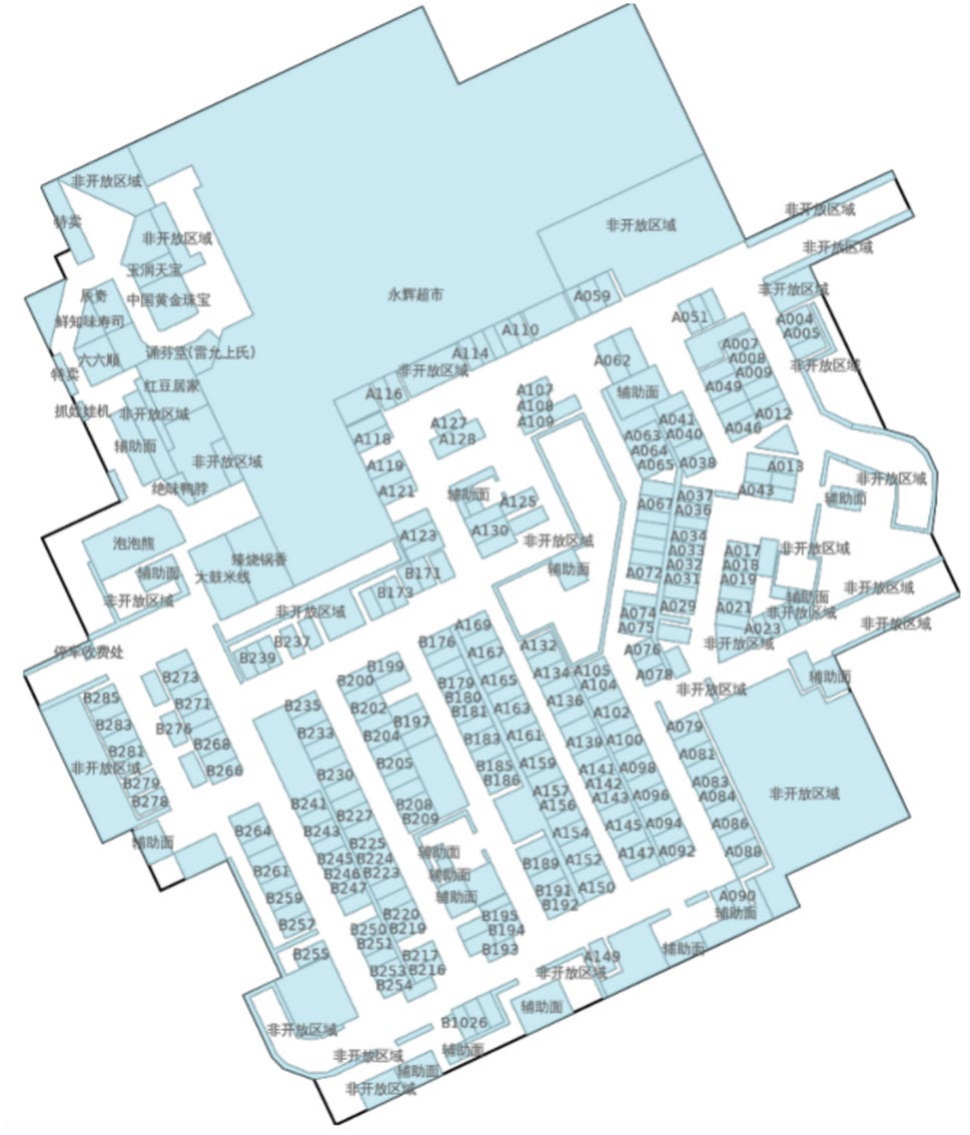
Sample floor plan from the microsoft research indoor location & navigation dataset.

Subsequently, to mitigate data scarcity and improve model generalization, a Sliding Window Augmentation technique was implemented. For every raw trajectory, multiple sub-sequences were generated (e.g., extracting [*A*, *B*] and [*A*, *B*, *C*] from a path [*A*, *B*, *C*]), drastically increasing the volume of training examples. The resulting augmented sequences were padded to a fixed length and partitioned into training, validation, and testing sets following an 80/10/10 ratio, with the target defined as the next discrete location token in the sequence. Figure 7 visualizes the complex layout of a representative shopping mall from the dataset, which serves as the physical domain for the trajectory generation and positioning tasks.

#### Model implementation

The sequence prediction model was implemented using a Long Short-Term Memory (LSTM) network, built with the TensorFlow Keras library^31^. The architecture was designed to capture the temporal dependencies within user movement patterns^11^. It begins with an Embedding layer that maps each integer-encoded location from the vocabulary of 91 unique places into a dense 128-dimensional vector representation. This layer is configured with mask_zero=True to ignore the padded values in the input sequences, ensuring the model learns only from valid location data. The output of the embedding layer is fed into an LSTM layer with 128 units and a recurrent dropout of 0.3 to mitigate overfitting on the sequential data. This is followed by a standard Dropout layer with a rate of 0.3 and a Dense hidden layer of 128 neurons with a ReLU activation function. The final Output layer is a Dense layer with 91 neurons one for each possible location and a softmax activation function to produce a probability distribution for the next predicted location. The model was compiled using the Adam optimizer and a categorical cross-entropy loss function. Training was performed for up to 50 epochs with a batch size of 256. To ensure robust training and prevent overfitting, two callbacks were utilized: an (EarlyStopping) callback with a patience of 5 epochs to halt training when the validation loss ceased to improve, and a (ModelCheckpoint) to save the weights of the best-performing model. Building on the previous work where sequence lengths from 2 to 12 were analyzed^32^, The test was done with a fixed input sequence length of 20 for this implementation. Shorter sequences, such as an initial path of only two locations, are automatically padded with zero values to match this fixed input dimension. Model performance was evaluated using top-1, top-3, and top-5 categorical accuracy metrics^33^ to assess its ability to rank the correct next location among its most likely predictions.

### Privacy-preserving training using federated learning

The final component of the machine learning pipeline addresses the privacy concerns associated with training on sensitive user location histories. To ensure that user data remains private, a Federated Learning (FL) approach was implemented. This method enables decentralized model training across multiple user devices, eliminating the need to upload private data to a central server. Instead, only model parameters are exchanged, ensuring privacy preservation throughout the learning process. This section outlines the data preparation for the federated setup and the implementation of the Federated Averaging (FedAvg) algorithm.

#### Federated dataset and preprocessing

Although the FL setup is based on the same cleaned location data from the StudentLife dataset, it required a distinct preprocessing pipeline tailored for distributed training. First, a global tokenizer was generated using all 90 unique location labels in the cleaned dataset. This ensures that both the central server and all clients share a consistent mapping of location names to integer identifiers. Using this unified vocabulary, the entire dataset was processed into sequences derived from 5,642 distinct user sessions. To emulate a realistic federated learning environment, the dataset was partitioned into two main parts. A centralized test set, comprising 15% of all sequences (16,469 sequences, each of length 8), was reserved exclusively for global evaluation. The remaining sequences were grouped according to the user_id attribute, resulting in 49 distinct local datasets, each corresponding to an individual client in the federated setup. Among these clients, 25 were used to pre-train the initial global model centrally, establishing a strong baseline, while the remaining 24 clients participated in the decentralized federated rounds.

To rigorously test the framework in the target retail domain, the Microsoft Research Indoor Location dataset was similarly adapted for federated simulation. Focusing on a specific target site (ID: 5cd56ba1e2acfd2d33b60373), the continuous (*x*, *y*) waypoints were simplified into 15 distinct spatial zones using K-Means clustering. This resulted in a vocabulary size of 16 (15 clusters plus padding). To address data scarcity in local partitions, a sliding window augmentation technique was applied to the raw traces. The resulting augmented trajectories were partitioned into a centralized pre-training set (50% of remaining sequences), a centralized test set (20% of all sequences), and a federated dataset sharded across 10 distinct clients to simulate a realistic distributed environment.

#### Federated averaging (FedAvg) implementation

The FL process was implemented as a simulation of the Federated Averaging (FedAvg) algorithm using TensorFlow, Keras, and NumPy^34^. This iterative, collaborative process is designed to improve a shared global model while ensuring that raw user data never leaves the client devices. The training begins with the server initializing a global LSTM model, which is first trained centrally using data from the initial 25 users. The trained model then serves as the baseline for subsequent federated rounds. Each training round begins with the selection of a random subset of clients to participate. The server then distributes the current global model weights to these clients, who proceed to train the model locally on their private datasets for several epochs, generating updated weights. Once local training is completed, the clients send their updated model weights back to the server. Finally, the server aggregates these updates using a weighted averaging scheme proportional to each client’s dataset size, producing a refined global model for the next round. This procedure, formally outlined in Algorithm 2, allows all clients to contribute to model improvement without exposing their private data.

**Algorithm 2:**
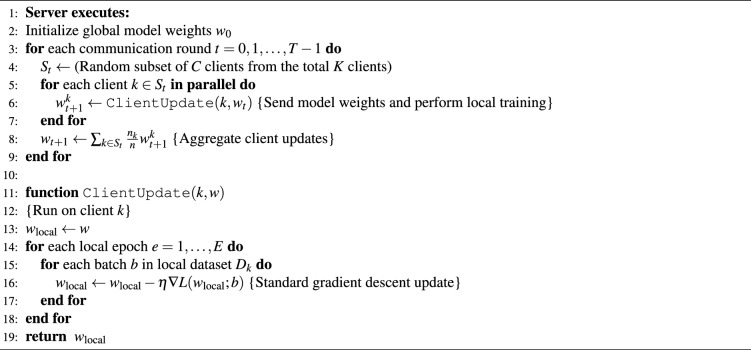
Federated averaging (FedAvg)

The core aggregation step (line 8 in Algorithm 2) updates the global model by computing a *weighted average* of the local models received from the participating clients. This aggregation is mathematically defined in Equation 1:

1 $$\begin{aligned} w_{t+1} = \sum _{k=1}^{C} \frac{n_k}{n} w_{t+1}^k \end{aligned}$$

Here, $w_{t+1}$ represents the global model weights after round $(t+1)$, while $w_{t+1}^k$ denotes the locally updated weights from client *k*. The weighting term $\frac{n_k}{n}$ ensures that clients with larger datasets contribute proportionally more to the aggregated model, where $n = \sum _{k=1}^{C} n_k$ is the total number of training samples across all participating clients. This approach balances model influence according to data availability, leading to a stable and representative global model.

For Local-Global Synchronization, synchronization is achieved through a synchronous round-based protocol. In each round t, the server waits for updates from all selected clients k in St. To mitigate the straggler effect (where slow clients delay the round), a timeout mechanism is enforced; clients failing to report within the window are dropped from the current round, ensuring the global model update remains timely.

### Mathematical formulation

o facilitate precise interpretation of the proposed framework, the core algorithms utilized for anomaly detection and sequence prediction are formally defined.

#### Anomaly detection models

**One-Class SVM (Proposed Gatekeeper):** The data integrity module utilizes a One-Class Support Vector Machine (OC-SVM) to map input Wi-Fi fingerprints into a high-dimensional feature space $\mathcal {F}$ via a kernel function $\phi : \mathcal {X} \rightarrow \mathcal {F}$. The objective is to find a hyperplane that separates the normal data from the origin with maximum margin. The optimization problem is defined as:

2 $$\begin{aligned} \min _{w, \xi , \rho } \frac{1}{2} ||w||^2 + \frac{1}{\nu l} \sum _{i=1}^{l} \xi _i - \rho \end{aligned}$$

Subject to $(w \cdot \phi (x_i)) \ge \rho - \xi _i$, with $\xi _i \ge 0$ for $i=1, \dots , l$. Here, $\nu$ is the regularization parameter controlling the upper bound of the outlier fraction (set to 0.12), and $\xi _i$ are slack variables.

**K-Nearest Neighbors (Baseline):** For the density-based baseline, the anomaly score $S_{KNN}(x)$ for a data point *x* is calculated as the average Manhattan distance ($L_1$ norm) to its *k* nearest neighbors ($\mathcal {N}_k(x)$) in the training set:

3 $$\begin{aligned} S_{KNN}(x) = \frac{1}{k} \sum _{x_j \in \mathcal {N}_k(x)} \sum _{d=1}^{D} |x^{(d)} - x_j^{(d)}| \end{aligned}$$

where *D* is the feature dimension. Points with a score exceeding the learned threshold are flagged as anomalies.

**Isolation Forest (Baseline):** The ensemble-based baseline isolates observations by randomly selecting a feature and a split value. The anomaly score *s*(*x*, *n*) is derived from the path length *h*(*x*) required to isolate a sample *x* across an ensemble of *T* trees:

4 $$\begin{aligned} s(x, n) = 2^{-\frac{E[h(x)]}{c(n)}} \end{aligned}$$

where *E*[*h*(*x*)] is the average path length and *c*(*n*) is the average path length of an unsuccessful search in a Binary Search Tree constructed from *n* samples. Shorter path lengths indicate a higher likelihood of being an anomaly.

#### LSTM for sequence prediction

The proactive navigation module models the probability of the next location $L_{t+1}$ given a sequence of visited locations $x = (x_1, x_2, \dots , x_t)$. The Long Short-Term Memory (LSTM) network processes this sequence through the following transition equations at each time step *t*:

5 $$\begin{aligned} i_t&= \sigma (W_i \cdot [h_{t-1}, x_t] + b_i) \end{aligned}$$

6 $$\begin{aligned} f_t&= \sigma (W_f \cdot [h_{t-1}, x_t] + b_f) \end{aligned}$$

7 $$\begin{aligned} o_t&= \sigma (W_o \cdot [h_{t-1}, x_t] + b_o) \end{aligned}$$

8 $$\begin{aligned} \tilde{C}_t&= \tanh (W_C \cdot [h_{t-1}, x_t] + b_C) \end{aligned}$$

9 $$\begin{aligned} C_t&= f_t \odot C_{t-1} + i_t \odot \tilde{C}_t \end{aligned}$$

10 $$\begin{aligned} h_t&= o_t \odot \tanh (C_t) \end{aligned}$$

Here, $i_t, f_t, o_t$ represent the input, forget, and output gates respectively; $C_t$ is the cell state, and $h_t$ is the hidden state passed to the dense output layer.

### Experimental configuration and reproducibility

To ensure the reproducibility of the proposed framework, the specific hyperparameters, training configurations, and environmental settings used across all experiments are detailed. The system implementation relies on Python 3.8, utilizing scikit-learn for anomaly detection and TensorFlow 2.x (Keras API) for deep learning and federated simulation.

#### Hyperparameter settings

Table 4 provides a comprehensive summary of the model configurations derived from the best-performing validation runs. For the LSTM sequence predictor, the Adam optimizer was utilized with an initial learning rate of 0.001, coupled with a ReduceLROnPlateau scheduler to dynamically adjust the learning rate. For anomaly detection, the One-Class SVM was tuned with a $\nu$ parameter of 0.12 to strictly bound the outlier fraction, utilizing a scaled RBF kernel.

**Table 4:** Summary of hyperparameters and experimental settings.

**Component**	**Parameter**	**Value/Setting**
**Anomaly detection**	**OC-SVM** Kernel	RBF (Radial Basis Function)
	**OC-SVM** Nu ($\nu$)	0.12
	**OC-SVM** Gamma	Scale ($1 / (n_{features} \cdot X.var())$)
	**KNN** Neighbors	$k=5$
	**KNN** Metric	Minkowski ($p=1$, Manhattan)
	**IsoForest** Estimators	100
	**IsoForest** Contamination	0.1
**Sequence prediction (LSTM)**	Input Sequence Length	20 time steps
	Embedding Dimension	128 (mask_zero=True)
	LSTM Units	128
	Dropout Layer	0.4
	Dense Layer Units	128 (ReLU activation)
	Optimizer	Adam ($\alpha _{init} = 0.001$) + LR Scheduler
	Batch Size	64
**Federated learning (FedAvg)**	Global Communication Rounds	4
	Total Client Pool	49 (StudentLife)/10 (Indoor Dataset)
	Clients per Round (*C*)	5 (Randomly selected)
	Local Epochs (*E*)	Tested $\{1, 10, 20\}$
	Aggregation Strategy	Synchronous Weighted Averaging
**Dataset splits**	Train/Validation/Test	80%/10%/10%

## Experimental results

This section presents a comprehensive evaluation of the machine learning components developed for the enhanced MetaWMS framework. The analysis is divided into three parts: first, a performance evaluation of the anomaly detection models; second, an assessment of the sequence prediction model’s accuracy; and third, an analysis of the privacy-preserving federated learning implementation.

### Performance of anomaly detection models

The primary objective of the data integrity module is to accurately distinguish between legitimate Wi-Fi signals (”normal”) and spurious signals (”anomalous”). To ensure robustness, the evaluation was conducted in two phases: first using controlled synthetic anomalies, and second using a real-world dataset.

#### Simulation with synthetic anomalies

To evaluate the initial effectiveness of the three implemented approaches K-Nearest Neighbors (KNN), One-Class SVM, and Isolation Forest a combined test set was utilized containing both normal workplace scans and synthetically generated anomalous signatures (simulating mobile hotspots).

The performance of each model was assessed using three standard classification metrics: accuracy, F1-score, and the Receiver Operating Characteristic (ROC) curve. Accuracy provides a measure of the overall correctness. The F1-score offers a robust measure particularly in cases of class imbalance^35^. Finally, the ROC curve illustrates the diagnostic ability of a classifier, with the Area Under the Curve (AUC) summarizing this performance^36^. Table 5 summarizes the performance comparison on the synthetic dataset. The superior performance of the One-Class SVM (AUC 0.963) is attributed to its ability to define a compact decision boundary around the normal Wi-Fi signal subspace. Unlike the Isolation Forest, which relies on random partitioning and struggled with the high variance of valid RSSI readings (resulting in a lower AUC of 0.822), the SVM effectively marginalized outliers. In practical terms, this 14% improvement in AUC translates to a significant reduction in false alarms, ensuring that legitimate users are not erroneously blocked from the AR navigation service while still filtering out mobile hotspot interference.

**Table 5:** Performance comparison of anomaly detection models (Synthetic data).

**Model**	**Accuracy (%)**	**F1-Score (%)**
KNN (Density-based)	86.8	64.2
OC-SVM	**93.5**	**78.6**
Isolation Forest	67.4	45.6

**Fig. 8:**
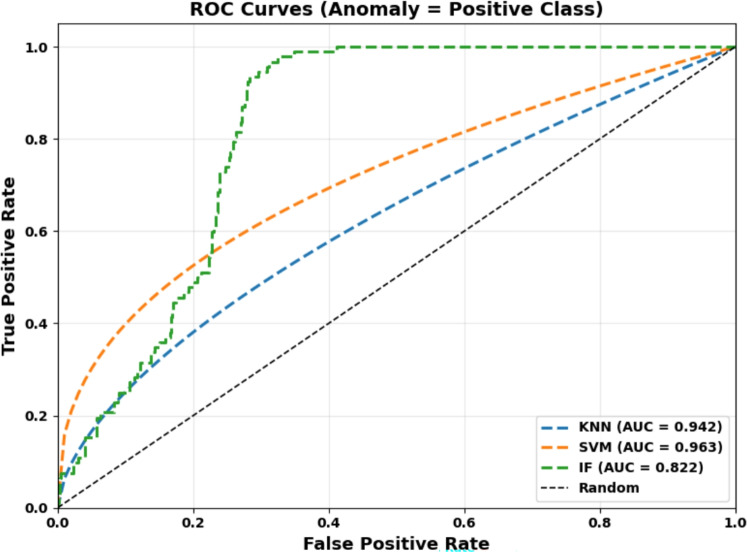
ROC-curve of different approaches (synthetic data).

The ROC curves in Fig. 8 illustrate the detection performance. The SVM model achieves the highest AUC value of 0.963, indicating superior discrimination between normal and anomalous samples. The KNN model follows closely with an AUC of 0.942. In contrast, the Isolation Forest performs noticeably worse (AUC 0.822), reflecting reduced reliability in this controlled setting.

#### Real-world validation (AWID3 dataset)

To validate the system against realistic, non-simulated irregularities, the models were further evaluated on the AWID3 dataset. This dataset introduces complex, real-world noise and ”Evil Twin/Rogue AP” patterns, which serve as a proxy for high-interference anomalies in public spaces.

**Fig. 9:**
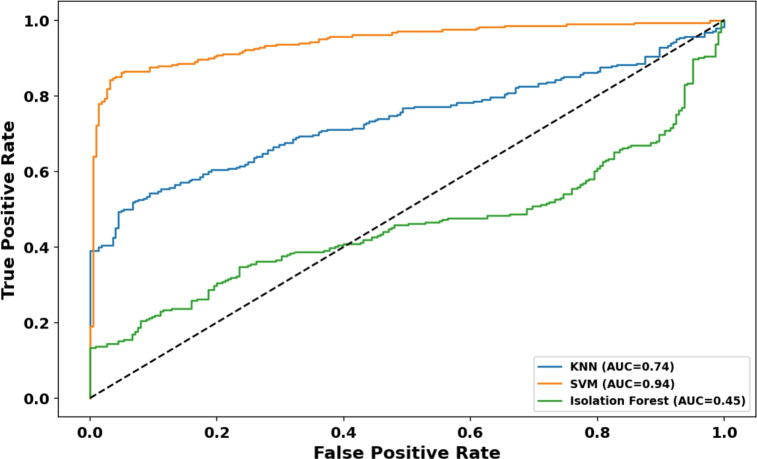
ROC-Curve of different approaches (Real-World AWID3 data).

Table 6 summarizes the performance metrics, renaming the target class to “Anomaly” to reflect the general filtering goal of the system. Additionally, Fig. 9 illustrates the Receiver Operating Characteristic (ROC) curves for the three models on this real-world dataset.

**Table 6:** Anomaly detection performance on real-world AWID3 dataset.

**Model**	**AUC**	**Sensitivity (Anomaly)**	**Specificity (Normal)**	**F1-Score (Anomaly)**
KNN	0.736	0.933	0.518	0.737
**One-Class SVM**	**0.945**	**0.951**	**0.861**	**0.895**
Isolation Forest	0.454	1.000	0.132	0.649

Consistent with the synthetic experiments, the One-Class SVM demonstrated superior performance, achieving the highest AUC of 0.945 (shown in orange in Fig. 9). It maintained an excellent balance between filtering anomalies (Sensitivity: 0.951) and preserving legitimate traffic (Specificity: 0.861). In contrast, the Isolation Forest model (green curve) suffered from extremely low specificity (0.132) and an AUC of 0.454, performing worse than a random classifier. This indicates a high false alarm rate, where the model incorrectly flagged the majority of normal scans as anomalies, likely due to the high variability of real-world RSSI values which the tree-based isolation method struggled to separate. These results confirm the One-Class SVM as the optimal choice for the data integrity module in diverse deployment scenarios.

### Performance of sequence prediction model

#### LSTM prediction evaluation

**Fig. 10:**
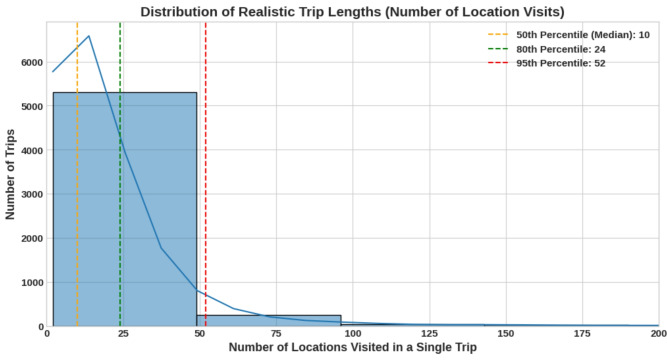
Distribution of realistic trip lengths.

The evaluation of the LSTM-based sequence prediction model focused on its ability to accurately forecast a user’s next location. A fixed input sequence length of 20 was selected for this implementation. This choice was informed by an analysis of user session lengths in the training data, as shown in Fig. 10. The data reveals that while the median trip length is 10 location visits, the 80th percentile is 24 visits and the 95th percentile is 52. A sequence length of 20 therefore provides a practical balance, capturing the majority of typical user sessions without being excessively long, which could introduce unnecessary computational complexity. To provide a comprehensive assessment, the LSTM model was evaluated on both the academic StudentLife dataset and the realistic Microsoft Research Indoor Location dataset. Table 7 presents the comparative results using top-*k* accuracy metrics. The performance disparity between the structured StudentLife dataset (Top-1: 53.76%) and the stochastic Microsoft dataset Indoor dataset (Top-1: 39.19%) is driven by the behavioral entropy of the domains. Workplace movements follow predictable schedules, whereas shopping trajectories are inherently random. However, the LSTM maintains a robust Top-5 accuracy (above 80%) in both scenarios. This indicates that while the model may not always pinpoint the exact next store, it successfully narrows down the user’s trajectory to the correct immediate zone, which is sufficient for pre-loading relevant AR content and reducing retrieval latency.

**Table 7:** Performance of the LSTM model: campus vs. shopping mall datasets.

**Metric**	**StudentLife (Campus)**	**Indoor location & navigation (shopping Mall)**
Top-1 Accuracy	53.76%	39.19%
Top-3 Accuracy	72.77%	71.79%
Top-5 Accuracy	81.26%	80.59%

**Fig. 11:**
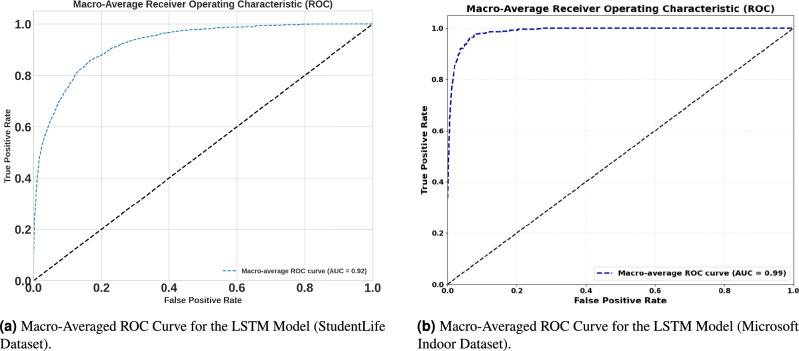
Comparison of macro-averaged ROC curves for LSTM models across both datasets.

To further assess the model’s classification performance across all 91 location classes, the macro-averaged Receiver Operating Characteristic (ROC) curve was investigated, shown in Fig. 11a. The macro-averaging technique computes the ROC curve for each class independently against all other classes and then averages the results, giving equal weight to each class regardless of its frequency^37^. This method is particularly useful for multi-class problems as it provides a fair assessment of the model’s ability to distinguish minority classes. The LSTM model achieved a macro-averaged Area Under the Curve (AUC) of 0.92. This high value indicates excellent discriminative capability across all location classes, confirming that the model has learned to effectively distinguish between the different possible next locations and is not biased towards only the most frequently visited places. Furthermore, the model’s discriminative ability on the shopping mall dataset was analyzed using the Receiver Operating Characteristic (ROC) curve. As illustrated in Fig. 11b, the model achieved an exceptional macro-average Area Under the Curve (AUC) of 0.99. This near-perfect separation suggests that despite the lower Top-1 accuracy caused by the high number of classes, the model assigns very low probabilities to incorrect locations, effectively filtering out irrelevant suggestions.

#### Comparative evaluation

To contextualize the performance of the deep learning approach, a comparative evaluation was first conducted using the StudentLife (Campus) dataset against traditional sequential pattern mining models from previous work^32^. The LSTM model was tested under the same experimental conditions to ensure a fair comparison. The results indicate that the LSTM model’s predictive accuracy surpasses that of both the CPT+ and CM-Spade models in this academic setting. However, its performance was found to be weaker than the Subseq model. This outcome highlights a fundamental trade-off between generalization and memorization. The Subseq model excels at identifying and recalling highly frequent, specific patterns from the training data, leading to higher accuracy on familiar sequences. In contrast, the LSTM network learns a more generalized representation of sequential patterns, which, while resulting in slightly lower accuracy on this specific dataset, makes it inherently more robust.

**Fig. 12:**
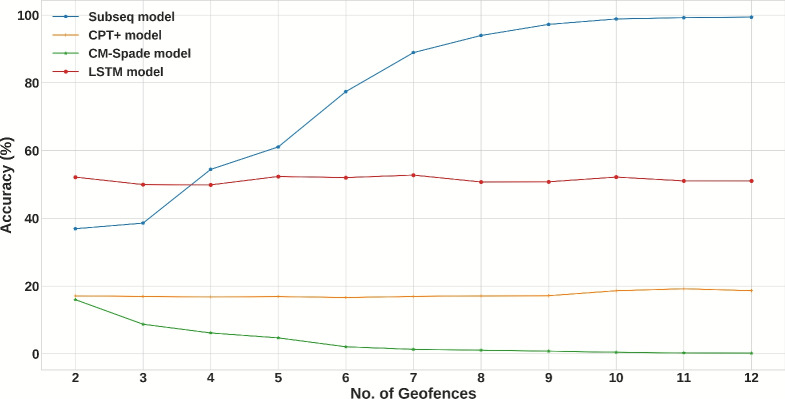
Different sequence prediction models accuracy (StudentLife dataset).

**Fig. 13:**
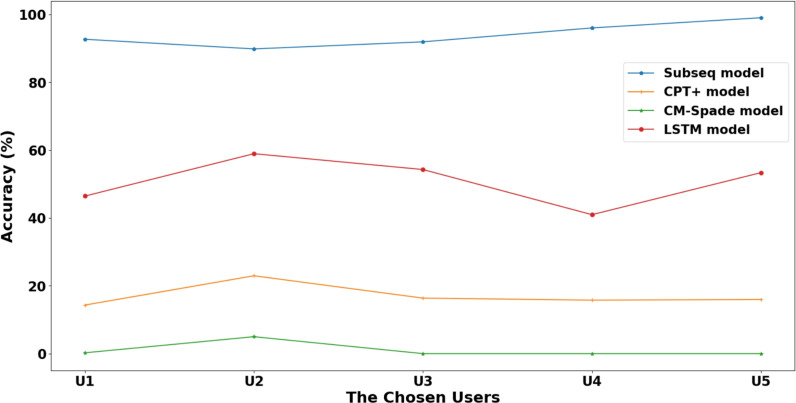
Accuracy for 8 Geofences as input (StudentLife dataset).

Figure 12 illustrates the model’s prediction accuracy on the StudentLife dataset as a function of the number of unique geofences. The trend shows that as the number of possible destinations increases, the prediction accuracy gracefully declines. Figure 13 highlights user-specific variability, indicating higher accuracy for users with consistent movement patterns. Tables 9, 10, and 11 provide the detailed breakdown of Top-5, Top-3, and Top-1 accuracies for the StudentLife users.

**Fig. 14:**
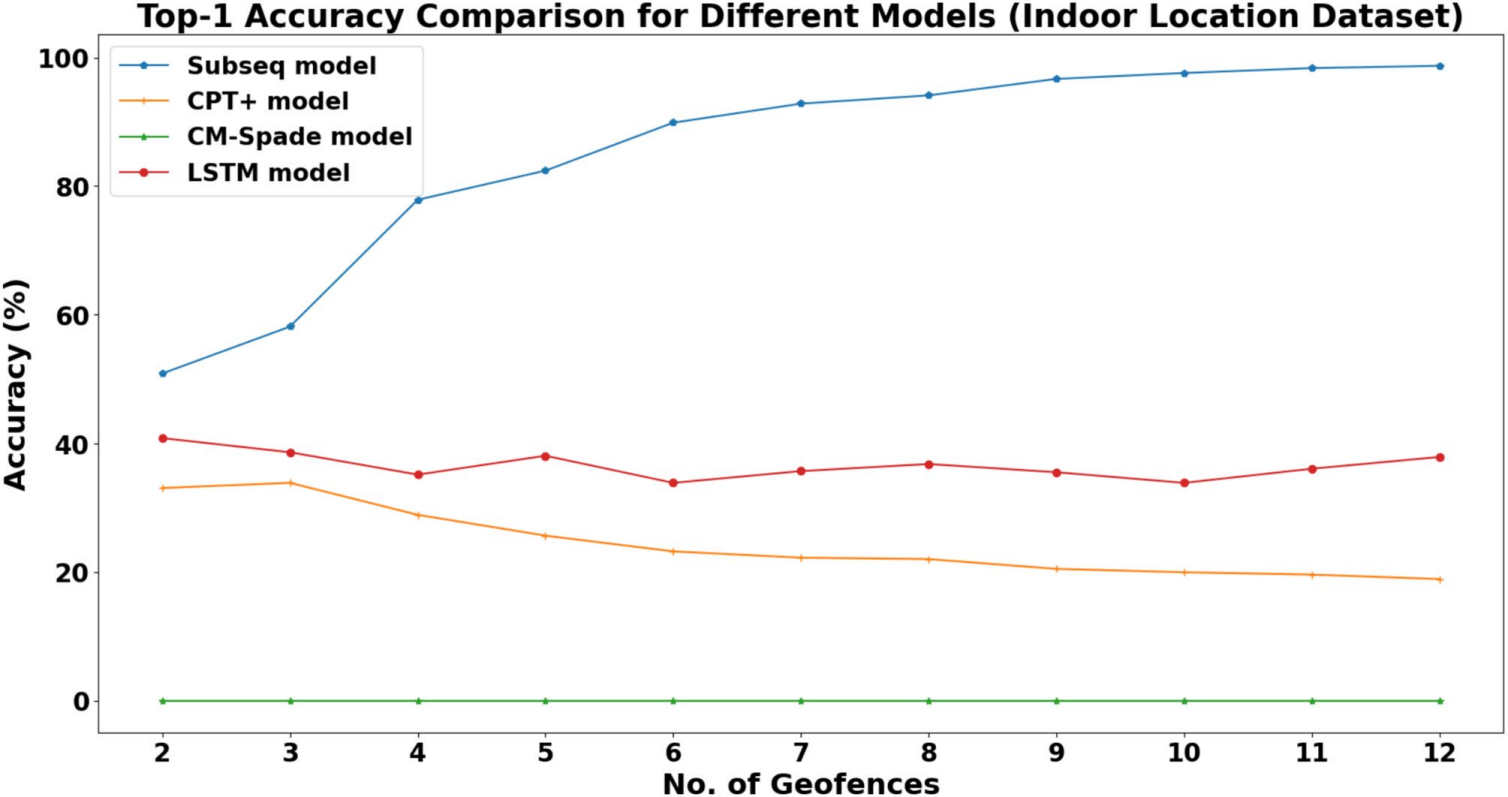
Top-1 accuracy comparison on the microsoft research indoor location dataset.

Validation on Retail Dataset (Microsoft): To rigorously stress-test the system in the target domain, the comparative analysis was extended to the Microsoft Research Indoor Location dataset. Figure 14 illustrates the Top-1 accuracy comparison in this complex retail environment. A critical finding here is the complete failure of the CM-SPADE algorithm, which yielded 0% accuracy across all sequence lengths. Unlike the structured campus data, the high sparsity and noise of real-world mall trajectories prevented CM-SPADE from mining statistically significant frequent patterns.

In contrast, the LSTM model demonstrated remarkable stability. While SubSeq still maintained high Top-1 accuracy due to strict memorization, the LSTM provided a reliable predictive baseline suitable for new user behaviors. o further quantify the LSTM’s robustness, an experiment was conducted varying the input sequence length from 2 to 12. The results, summarized in Table 8, show that the model maintains high usability even with short context windows. Notably, the Top-5 accuracy remains consistently robust, peaking at 80.59% for a sequence length of 3, confirming the system’s ability to offer relevant navigational suggestions to shoppers.

**Table 8:** LSTM performance vs. sequence length (indoor location & navigation retail dataset).

**Seq. Length**	**Top-1 (%)**	**Top-3 (%)**	**Top-5 (%)**
2	**40.84**	**71.25**	80.04
3	38.64	70.33	**80.59**
4	35.16	65.57	77.66
5	38.10	69.78	77.84
6	33.88	65.20	77.47
7	35.71	67.58	76.37
8	36.81	68.86	78.21
9	35.53	63.55	76.92
10	33.88	68.50	79.12
11	36.08	69.60	80.22
12	37.91	69.41	80.04

**Table 9:** LSTM top-5 accuracy (StudentLife dataset).

User	Algorithm	No. of Geofences										
		2	3	4	5	6	7	8	9	10	11	12
User0	LSTM	72.55	75.74	65.79	79.10	78.99	68.00	69.29	57.34	71.06	65.81	62.40
User1		80.50	80.42	82.77	76.03	79.92	85.60	81.41	85.22	85.25	86.87	84.39
User2		76.79	77.95	66.56	73.64	69.70	70.07	77.61	80.00	76.14	62.18	75.19
User3		67.50	70.00	79.17	71.70	63.24	63.64	62.30	75.00	95.24	81.58	96.43
User4		81.65	78.46	78.26	77.93	77.37	81.39	80.16	82.28	82.63	76.87	80.17

**Table 10:** LSTM top-3 accuracy (StudentLife dataset).

User	Algorithm	No. of Geofences										
		2	3	4	5	6	7	8	9	10	11	12
User 0	LSTM	65.36	68.64	56.58	73.13	71.21	61.60	61.42	51.38	64.26	63.25	57.60
User 1		80.34	74.83	78.15	71.29	73.23	80.63	76.42	80.52	82.60	79.00	80.35
User 2		70.36	70.26	60.45	68.64	57.30	61.31	68.40	71.22	67.97	59.24	69.63
User 3		52.50	60.00	56.25	62.26	58.82	54.55	54.10	65.00	71.43	52.63	89.29
User 4		73.73	70.29	68.03	71.14	70.47	69.90	71.78	74.81	72.34	69.64	72.35

**Table 11:** Comparison of top-1 accuracy across algorithms (StudentLife dataset).

User	Algorithm	No. of Geofences										
		2	3	4	5	6	7	8	9	10	11	12
User 1	LSTM	49.02	50.89	43.42	58.96	52.14	48.80	46.46	44.04	54.89	50.43	45.60
User 2	LSTM	57.28	58.74	54.46	53.94	54.33	58.90	58.96	67.13	61.65	56.32	61.46
User 3	LSTM	55.71	52.31	48.55	54.09	50.96	47.45	54.29	51.71	54.25	49.16	52.59
User 4	LSTM	32.50	35.00	35.42	45.28	39.71	41.82	40.98	37.50	38.10	39.47	46.43
User 5	LSTM	50.00	48.98	46.55	50.59	51.08	48.87	53.37	52.05	50.76	46.99	48.70
User 1	SubSeq	37.90	41.09	57.76	63.84	80.25	89.26	92.71	96.58	99.32	99.59	100.00
User 2	SubSeq	47.37	46.50	56.02	60.71	72.34	83.78	89.87	93.50	96.03	96.39	96.70
User 3	SubSeq	32.65	34.97	52.07	59.24	77.79	88.64	91.95	96.49	98.91	100.00	100.00
User 4	SubSeq	31.41	33.47	46.56	51.73	67.89	86.83	96.05	99.45	99.77	100.00	100.00
User 5	SubSeq	35.18	36.74	59.60	69.67	88.56	95.86	99.06	100.00	100.00	100.00	100.00
User 1	CM-SPADE	17.60	8.71	7.25	2.11	0.55	0.00	0.24	0.00	0.00	0.00	0.00
User 2	CM-SPADE	27.80	21.30	16.35	12.39	9.21	6.31	4.99	3.83	2.18	1.20	0.89
User 3	CM-SPADE	15.31	6.87	4.02	1.15	0.00	0.00	0.00	0.00	0.00	0.00	0.00
User 4	CM-SPADE	12.29	6.56	3.08	7.80	0.55	0.17	0.00	0.00	0.00	0.00	0.00
User 5	CM-SPADE	6.82	0.15	0.20	0.10	0.00	0.00	0.00	0.00	0.00	0.00	0.00
User 1	CPT+	17.48	15.73	15.23	14.33	12.91	14.52	14.32	15.66	17.51	18.21	15.07
User 2	CPT+	24.39	24.18	23.14	23.24	22.45	23.01	22.96	22.0	23.01	22.11	23.05
User 3	CPT+	14.02	15.23	14.26	15.89	15.90	15.93	16.37	16.73	20.0	20.63	19.23
User 4	CPT+	14.72	15.10	15.78	15.70	15.99	15.60	15.77	16.03	17.63	16.44	17.16
User 5	CPT+	14.30	14.30	15.48	15.26	15.69	15.51	15.96	15.23	14.85	18.46	18.60

### Performance of federated learning

The final phase of the experimental evaluation focused on quantifying the performance of the privacy-preserving Federated Learning (FL) implementation. The primary objectives were to assess the convergence of the global sequence prediction model in a decentralized setting and to analyze the impact of local computation on model accuracy.

#### Campus environment evaluation (StudentLife)

**Fig. 15:**
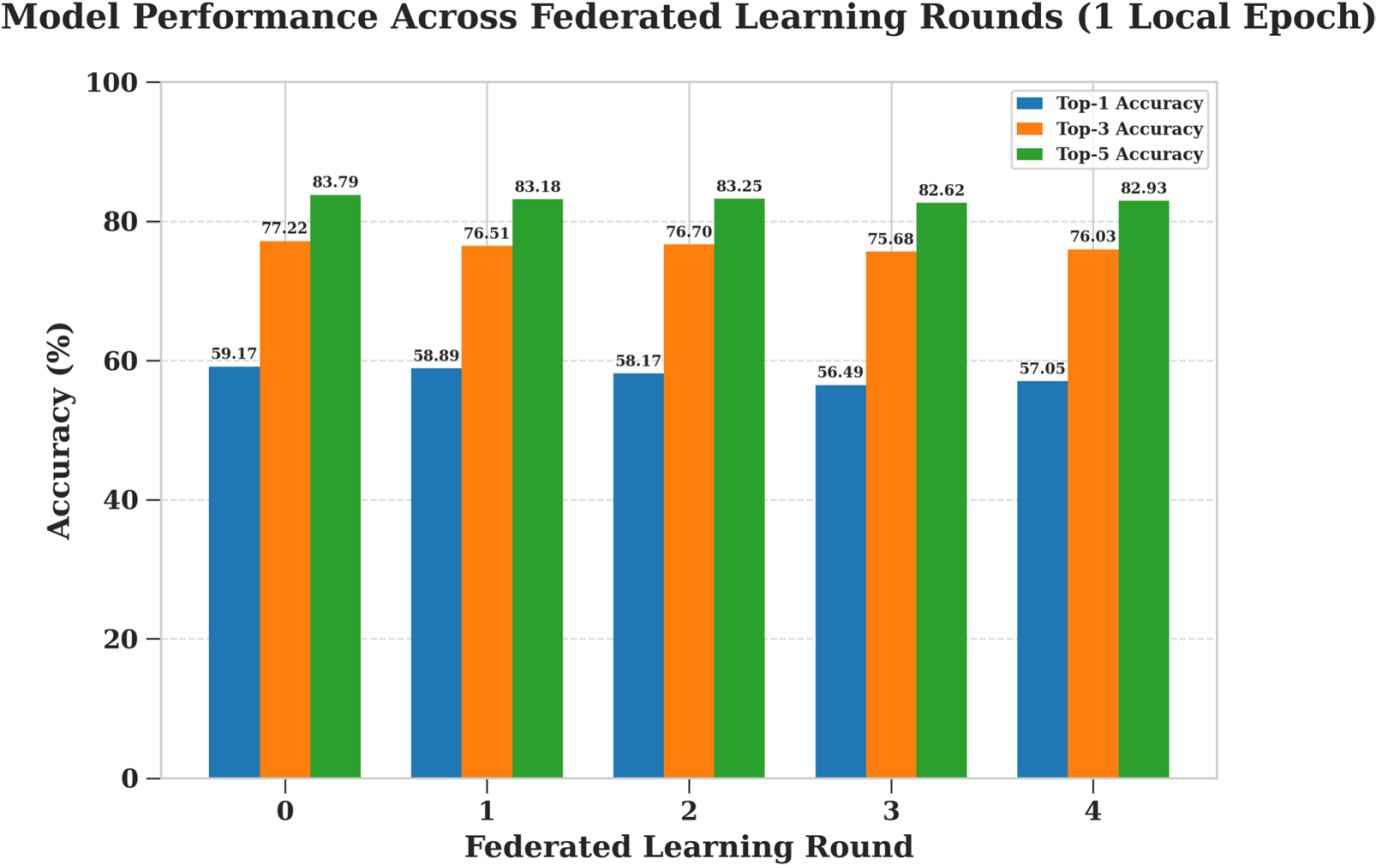
Model evaluation with (1) local training epoch (StudentLife).

**Fig. 16:**
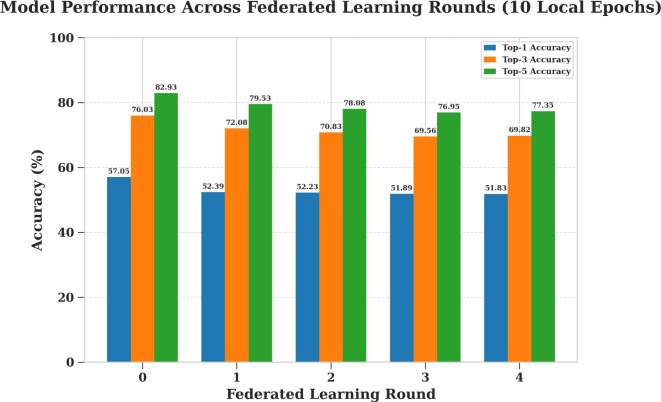
Model evaluation with (10) local training epoch (StudentLife).

**Fig. 17:**
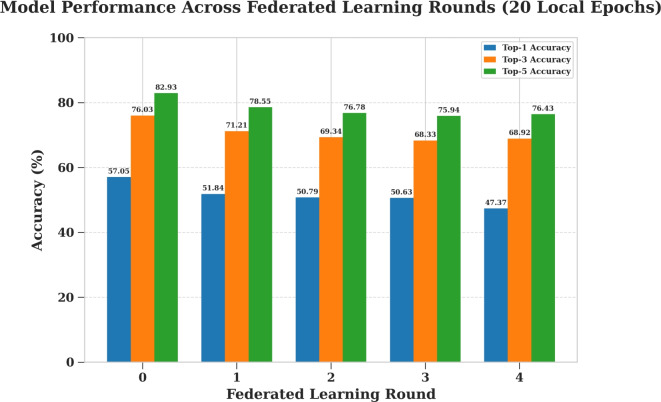
Model evaluation with (20) local training epoch (StudentLife).

To investigate the trade-off between communication efficiency and model performance using the StudentLife dataset, a 4-round training process was conducted three separate times, varying the amount of local computation performed by each client in a given round. Specifically, the tested scenarios involved clients training locally for 1, 10, and 20 epochs before sending their weight updates back to the server. The results of these experiments, detailing the progression of the top-k accuracies for each local epoch setting, are presented in Figures 15, 16, and 17 respectively.

Table 12 summarizes the To/p-1, Top-3, and Top-5 prediction accuracies across five communication rounds for different local training epochs. As shown, models trained with a single local epoch maintain relatively consistent accuracy across rounds, while those trained with more local epochs exhibit a gradual decline, especially after the initial rounds. Although the LSTM model demonstrates lower overall accuracy compared to the other algorithms, it was selected due to its compatibility with federated learning, allowing for the aggregation of model weights across distributed clients without sharing raw data.

**Table 12:** Top-1, Top-3, and Top-5 accuracies across rounds (StudentLife dataset).

Local Epochs	Accuracy	No. of rounds				
		0	1	2	3	4
1	Top-1	59.17	58.89	58.17	56.49	57.05
	Top-3	77.22	76.51	76.70	75.68	76.03
	Top-5	83.79	83.18	83.25	82.62	82.93
10	Top-1	57.05	52.39	52.23	51.89	51.83
	Top-3	76.03	72.08	70.83	69.56	69.82
	Top-5	82.93	79.53	78.08	76.95	77.35
20	Top-1	57.05	51.84	50.79	50.63	47.37
	Top-3	76.03	71.21	69.34	68.33	68.92
	Top-5	82.93	78.55	76.78	75.94	76.43

#### Retail environment evaluation (indoor location & navigation)

The federated learning evaluation was extended to the retail environment to assess performance on the clustered Microsoft dataset. This simulation utilized a pool of 10 clients, with 5 participating in each of the 4 communication rounds. Each client performed 20 local epochs of training using an LSTM architecture optimized for the 16-class vocabulary (15 spatial clusters + padding).

**Fig. 18:**
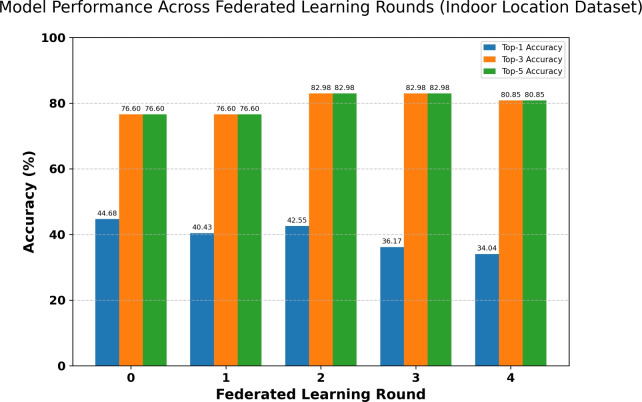
Model performance across federated learning rounds (indoor location dataset).

As illustrated in Fig. 18, the model began with a centralized pre-training baseline (Round 0) achieving 44.68% Top-1 accuracy and 76.60% Top-5 accuracy. Throughout the federated rounds, the Top-5 accuracy demonstrated robustness, peaking at 82.98% in Rounds 2 and 3, and concluding at 80.85% in Round 4. Conversely, the Top-1 accuracy exhibited a decline, dropping to 34.04% by the final round. This divergence highlights the challenge of non-IID data distributions in federated settings, where local updates may drift from the global optimum for specific predictions. However, the high and stable Top-5 accuracy confirms that the system remains highly effective at narrowing down the user’s likely next zone, maintaining utility for the recommendation engine while preserving user privacy.

### Computational efficiency and edge deployability

To assess the feasibility of deploying the proposed models on resource-constrained edge devices (e.g., smartphones in the MetaWMS ecosystem), a rigorous evaluation of computational efficiency was conducted. Key metrics included *Inference Latency* (time per sample), *Model Size* (storage footprint), *FLOPs* (floating-point operations), and estimated energy consumption. The evaluation simulated a production environment by converting the deep learning models to the TensorFlow Lite (TFLite) format^28^ with standard optimizations.

#### Anomaly detection model (One-Class SVM)

The One-Class SVM, selected for the data integrity module, demonstrated exceptional lightweight characteristics suitable for continuous background execution. When serialized using the scikit-learn library^23^, the model occupied a minimal storage footprint of 15.34 KB. The average inference latency was measured at 0.1615 ms per sample on a standard CPU thread. This negligible latency ensures that the anomaly filter operates as a transparent gatekeeper, introducing virtually no overhead before the core geofencing logic.

#### Sequence prediction model (LSTM)

To validate the deployability of the sequence prediction module, the trained LSTM network was converted to a quantized TensorFlow Lite (TFLite) model^28^. As detailed in Table 13, this optimization process resulted in a 90.13% reduction in model size, compressing the original 3.2 MB Keras model to just 317.16 KB.

Comprehensive profiling of the edge-optimized model revealed a computational complexity of approximately 0.42 MFLOPs (Mega Floating-Point Operations) per inference. Based on standard industry estimates for mobile ARM processors (approx. 5 nJ per FLOP^38^), the theoretical energy consumption is estimated at 2.11 mJ per prediction. The model achieved a peak RAM usage of 60.54 KB during inference and an average latency of 0.1493 ms, enabling a theoretical throughput of over 6,600 samples per second. These metrics confirm that the predictive navigation system can run entirely on-device with minimal impact on battery life or system resources.

**Table 13:** Computational efficiency metrics for edge deployment.

**Model**	**Format**	**Size (KB)**	**Latency (ms)**	**FLOPs**
Data Integrity (OC-SVM)	Scikit-learn	15.34	0.1615	N/A
Sequence Prediction (LSTM)	TFLite (Quantized)	317.16	0.1493	0.42 M

## Discussion

The experimental results presented in this study validate the proposed framework’s ability to bridge the gap between physical location sensing and intelligent, privacy-preserving user guidance. By integrating anomaly detection, sequence prediction, and federated learning, the system addresses key challenges in modern IoT environments: signal noise, predictive utility, and data privacy.

### Robustness against environmental noise

A critical finding of this research is the necessity of the anomaly detection “gatekeeper” in dynamic public spaces. The comparison between the synthetic and real-world AWID3 datasets highlighted the fragility of standard classifiers when exposed to transient signals like mobile hotspots. The One-Class SVM’s superior performance (AUC 0.945) on the AWID3 dataset confirms that boundary-based methods are more effective than density-based (KNN) or isolation-based methods for high-dimensional Wi-Fi fingerprints. This preprocessing step is not merely an optimization but a requirement for reliable indoor geofencing; without it, the transient nature of “Evil Twin” or hotspot signals would render the subsequent location classification unstable.

### Trade-offs in sequence prediction

The comparative analysis of the LSTM model against traditional sequential pattern mining algorithms (SPM) reveals a fundamental trade-off between *memorization* and *generalization*. While the SubSeq algorithm achieved higher accuracy on the structured StudentLife dataset by effectively memorizing frequent paths, it faltered in the complex, noisy environment of the retail dataset. In contrast, the LSTM model demonstrated remarkable stability, maintaining a Top-5 accuracy of over 80% even on the sparse Microsoft dataset where algorithms like CM-SPADE failed completely. This suggests that for real-world deployment, where user behaviors are diverse and less repetitive than campus routines, the deep learning approach offers superior utility.

### Privacy and communication trade-offs

The proposed architecture balances privacy and communication efficiency through the Federated Averaging strategy. By keeping raw trajectory data on-device, the privacy risk of centralized logging is eliminated. However, this introduces a communication overhead of transmitting model weights (approx. 317 KB) per round. Experimental results (Table 10) demonstrate that setting local epochs to E=1 yields the highest stability, but increasing E to 20 significantly reduces communication frequency by allowing the model to converge in fewer global rounds. This represents a tunable trade-off: critical security environments may favor higher E to minimize network exposure, while navigation-critical apps require lower E for rapid map updates.

### Hardware constraints and deployment feasibility

While the visual-inertial odometry (VIO) used for AR rendering remains dependent on the underlying hardware (e.g., ARCore stability) as noted in^39^, experimental benchmarks by Feigl et al.^40^ indicate that the average positional drift remains within acceptable bounds for navigation tasks. Consequently, the core logic for anomaly filtering and next-location prediction is lightweight enough to run continuously in the background without draining battery resources.

### Comparison with coordinate-based localization (FEDHIL)

A distinction must be drawn between the coordinate-based localization utilized in SOTA frameworks like FedHIL^13^ and the zone-based classification employed in this work. FedHIL focuses on minimizing the Root Mean Square Error (RMSE) of spatial coordinates (x, y), achieving high precision at the cost of increased model complexity and sensitivity to device heterogeneity. In contrast, MetaSNS prioritizes semantic accuracy (e.g., correctly identifying ”Zone: Zara Store”). Experimental results indicate that while the proposed geofencing approach simplifies the spatial output, it achieves a high classification accuracy of 95% with significantly lower computational overhead than regression-based methods. For retail recommendation engines, this semantic ”room-level” granularity is often more actionable and privacy-preserving than continuous coordinate tracking.

### Domain generalizability: campus vs. retail mobility

A key concern in developing navigational models is the transferability of mobility patterns across different domains. While the StudentLife dataset^29^ serves as a robust proxy for workplace environments (MetaWMS) characterized by structured, repetitive routines and long dwell times–it does not fully capture the stochastic nature of shopping behavior. To ensure domain applicability for the MetaSNS shopping framework, the Microsoft Research Indoor Location dataset^30^ was integrated, which captures dense, multi-floor retail trajectories.

The experimental results reveal a divergence in predictive performance: the LSTM achieves higher Top-1 accuracy on the structured Campus dataset (53.76%) compared to the dynamic Retail dataset (39.19%). This confirms that shopping mall mobility is inherently more entropic and less predictable than workplace routines. However, the consistent Top-5 accuracy across both domains ($\approx 80\%$) demonstrates that the proposed architecture generalizes well. By training on both datasets, the system’s dual applicability is validated: supporting highly predictable routine-based monitoring for workplaces and flexible, suggestion-based navigation for retail environments.

### Comparative analysis with state-of-the-art baselines

To contextualize the performance of the MetaSNS framework, the results are contrasted with both traditional sequential pattern mining algorithms (SubSeq, CPT+) and recent deep learning approaches identified in the literature.

Rationale for Baseline Selection: Traditional baselines (SubSeq, CPT+, CM-SPADE) were selected to represent the class of memorization-based algorithms, which excel in highly repetitive environments. Conversely, recent baselines like Transformer Encoders^9^ and FedHIL^13^ represent the state-of-the-art in generalization-based and coordinate-based methods, respectively.

Performance Analysis: While Transformer-based approaches^9^ theoretically offer superior long-range dependency capture, their computational cost (often requiring GPUs) renders them unsuitable for continuous background execution on consumer mobile devices. he proposed LSTM implementation strikes a critical balance: it achieves 80.59% Top-5 accuracy–comparable to the semantic utility of complex Transformers–while maintaining an inference latency of just 0.14 ms (Table 13), ensuring battery viability.

Furthermore, compared to FedHIL’s coordinate regression^13^, the proposed zone-based classification is more robust to the ”device heterogeneity” problem. Regression models often suffer from high variance when trained on RSSI data from diverse smartphone chipsets. By simplifying the output space to discrete geofences and filtering noise via OC-SVM (AUC 0.945), the system achieves higher stability in real-world deployment, albeit at a lower spatial resolution.

### Challenges and mitigation strategies

The deployment context introduces specific challenges regarding temporal coupling and spatio-contextual representation. Unlike independent image classification tasks, user mobility data exhibits strong temporal coupling. This is mitigated by the stateful nature of the LSTM architecture, which maintains a local hidden state across the sequence, allowing the model to internalize temporal dependencies before weight aggregation. Regarding spatio-contextual representation, the abstraction of raw coordinates into semantic tokens (geofences) reduces the dimensionality of the feature space, making the global model less sensitive to minor spatial variations across heterogeneous devices. Finally, scalability under dynamic client participation is managed through the random subset selection protocol (C=5), which prevents server bottlenecks and ensures the global model does not overfit to a specific subset of active users.

Future research will focus on three strategic enhancements to address these gaps. First, the fusion of multimodal sensor data specifically Bluetooth Low Energy (BLE) and Ultra-Wideband (UWB) will be explored to improve fine-grained positioning accuracy beyond room-level geofencing. Second, advanced federated strategies such as asynchronous aggregation and differential privacy will be investigated to further optimize communication efficiency and security. Finally, a large-scale, longitudinal user study in a live mall environment is planned to quantitatively assess the impact of AR guidance on shopper dwell times and satisfaction, thereby solidifying the role of intelligent navigation in the next generation of smart, privacy-preserving metaverse environments.

## Conclusion

The convergence of the Metaverse with physical IoT environments represents a paradigm shift in how workspaces and retail venues are experienced. This research established that a robust, privacy-preserving navigational framework is not only feasible but essential for fostering trust in these hyper-connected ecosystems. By integrating a multi-stage machine learning pipeline, the MetaSNS framework successfully addresses the twin challenges of signal volatility and data privacy.

Empirically, the introduction of a One-Class SVM gatekeeper proved critical for identifying spurious ”rogue” signals. Validated against the industry-standard AWID3 benchmark, this module achieved an AUC of 0.945, ensuring that subsequent localization relies solely on high-fidelity data. Furthermore, the transition from reactive detection to proactive guidance was realized through an LSTM sequence predictor, which maintained a Top-5 accuracy of 80.59% even within the stochastic mobility patterns of the Microsoft Research Indoor Location dataset. Crucially, the deployment of Federated Learning demonstrated that user privacy need not come at the cost of utility; a marginal accuracy trade-off of 2–5% was sufficient to keep sensitive trajectory data entirely on-device.

While limitations remain regarding the dependence on Wi-Fi fingerprinting and potential variations in real-world Federated Learning convergence times, the proposed architecture offers a scalable foundation. By fusing semantic geofencing with decentralized intelligence, this work paves the way for the next generation of secure, context-aware Metaverse environments.

## Data Availability

The results of this study are based on three publicly accessible datasets and one proprietary dataset. The workplace mobility patterns were analyzed using the StudentLife dataset^41^. Real-world anomaly detection was evaluated using the Aegean Wi-Fi Intrusion Dataset 3 (AWID3)^24^. The retail navigation framework was validated using the Indoor Location & Navigation dataset provided by Microsoft Research^30^. Additional datasets generated during the study, including the specific campus Wi-Fi fingerprints and synthetic anomaly sets, are available from the corresponding author upon reasonable request.
